# Rethinking Synthetic Berberine in Nutraceuticals: Nitrosamine Risks, Regulatory Oversight, and Safer Alternatives

**DOI:** 10.3390/molecules30214274

**Published:** 2025-11-03

**Authors:** Anil Kumar Meher, Akli Zarouri, Manish Kumar

**Affiliations:** 1 Department of Bioproducts and Biosystems Engineering, University of Minnesota, St. Paul, MN 55108, USA; azarouri@umn.edu; 2 Amity Institute of Biotechnology, Amity University Noida, Noida 201 303, India; mkumar32@amity.edu

**Keywords:** berberine hydrochloride, synthetic nutraceuticals, nitrosamine impurities, residual solvents, ICH Q3C (R8), ICH M7, DSHEA 1994

## Abstract

Berberine hydrochloride, a bioactive isoquinoline alkaloid with metabolic, lipid-lowering, and antimicrobial properties, is increasingly synthesized for nutraceutical applications due to its high yield and, therefore, cost-effectiveness. However, synthetic production can raise significant safety concerns, particularly regarding potentially toxic residuals generated during chemical process. A primary concern is the formation of nitrosamines, a class of genotoxic impurities associated with the use of secondary amines, nitrites, and strong acids in synthetic processes. This article examines the reported synthetic routes for berberine hydrochloride, with emphasis on the use of toxic reagents and intermediates that, if not completely consumed or effectively removed during synthesis process, may persist as impurities in the final product. In contrast, berberine obtained with aqueous botanical extraction avoids such hazardous synthetic precursors and retains co-occurring alkaloids, offering a cleaner safety profile and potential synergistic benefits.

## 1. Introduction

**Figure 1:**
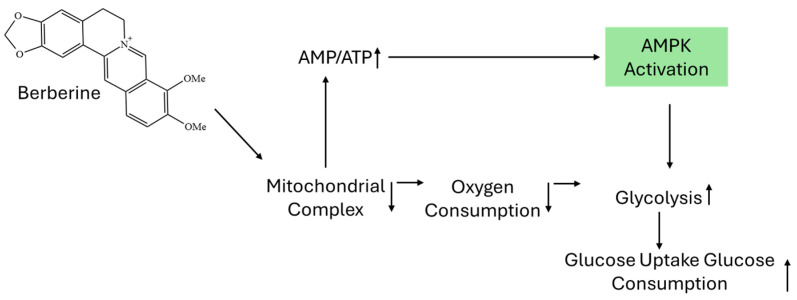
Mechanism of berberine-induced AMPK activation and metabolic effects.

Berberine hydrochloride, a quaternary isoquinolinium alkaloid [1], is widely recognized for its pharmacological effects, particularly in the management of type 2 diabetes, hyperlipidemia, and microbial infections [2]. It occurs naturally in several medicinal plants, most notably *Berberis aristata*, *Coptis chinensis*, and *Hydrastis canadensis*, and has a longstanding history of use in both Eastern and Western herbal medicine traditions [3]. Berberine’s anti-diabetic activity is primarily attributed to the activation of AMP-activated protein kinase (AMPK) [4], a central regulator of cellular energy homeostasis, as shown in Figure 1. AMPK activation enhances glucose uptake, improves insulin sensitivity, and suppresses hepatic gluconeogenesis. In addition to its direct effect on AMPK, berberine inhibits mitochondrial respiratory complex I, leading to reduced oxygen consumption and an elevated intracellular AMP/ATP ratio, which further stimulates AMPK activation [5]. This cascade promotes increased glycolysis and facilitates glucose uptake and utilization in peripheral tissues [6].

Beyond its metabolic effects, recent research highlights berberine’s potent antioxidant potential and its capacity to strengthen endogenous cellular defenses, thereby mitigating oxidative stress and protecting against ROS-induced damage. Oxidative stress plays a central role in the development of metabolic, cardiovascular, inflammatory, and neurodegenerative disorders, and compounds capable of modulating redox balance hold significant therapeutic promise.

Berberine exhibits multifaceted antioxidant activity by enhancing key enzymatic defenses, superoxide dismutase (SOD), catalase (CAT), and glutathione peroxidase (GPx), and by activating signaling pathways such as AMP-activated protein kinase (AMPK) and nuclear factor erythroid 2—related factor 2 (Nrf2)

These pathways regulate cellular energy metabolism and antioxidant gene expression, respectively, thereby maintaining redox homeostasis and protecting cells from oxidative injury. Several studies have reported that berberine increases SOD, CAT, and GPx activity in the liver, brain, and heart, leading to reduced lipid peroxidation and restoration of glutathione levels [7]. Activation of AMPK improve mitochondrial function, limits ROS generation, and modulates cellular metabolism, while Nrf2 activation induces transcription of cytoprotective and phase II detoxifying enzymes [8]. In models of neuronal injury, berberine has been shown to attenuate ferroptosis by inducing AMPK phosphorylation and upregulating Nrf2 and HO-1, ultimately reducing lipid peroxidation and iron accumulation [9].

In addition to these indirect effects, berberine demonstrates direct antioxidant action through free radical scavenging and transition-metal chelation. Its molecular structure enables electron or hydrogen donation to neutralize reactive radicals such as hydroxyl and superoxide species, thereby preventing oxidative modifications of lipids, proteins, and nucleic acids [10]. Furthermore, berberine’s chelation of metal ions such as Fe^2+^ and Cu^2+^ reduces catalytic ROS generation (e.g., via Fenton reactions), complementing enzymatic and signaling-mediated mechanism. This combination of actions, enhancing enzymatic antioxidant systems, modulating redox signaling, scavenging radicals, and chelating pro-oxidant metals, renders berberine a comprehensive antioxidant agent with broad biomedical relevance [11].

Despite its strong pharmacological properties, berberine’s therapeutic use is limited by poor aqueous solubility, low intestinal absorption, and extensive first-pass metabolism. Recent formulation advances have addressed these challenges, improving its bioavailability and antioxidant efficacy. For instance, phytantriol-based liquid crystalline nanoparticles loaded with berberine reduced inflammation and ROS generation in lipopolysaccharide-stimulated macrophages [12]. Lipid-based nanoparticles of berberine hydrochloride enhanced pancreatic β-cell function by modulating the Nrf2/NF-κB pathway in diabetic rats [13]. Likewise, “gelled-oil” nanocarriers improved berberine’s stability and controlled its release [14], while nanomicelles demonstrated superior in vitro and in vivo antioxidant and anti-inflammatory effects compared to free berberine, with increased activity of SOD, CAT, and GPx and reduced markers of oxidative damage in brain tissue [15].

Together, these findings indicate that berberine combats oxidative stress through multiple complementary mechanisms, enhancing antioxidant enzyme activity, activating AMPK and Nrf2 pathways, scavenging free radicals, and chelating metal ions while advances in nanoparticle, liposomal, phytosomal, and micellar delivery systems have significantly improved its solubility, absorption, and in vivo efficacy. These dual developments in mechanistic understanding and formulation optimization underscore berberine’s promising potential as a natural antioxidant therapeutic, warranting further molecular, pharmacokinetic, and clinical investigation.

Of course, it is important to clarify the interest in synthetic berberine. Synthetic approaches may allow for improved stability, consistency, and potency compared to natural extracts. However, in recent years, growing concerns have emerged over the adulteration of “all-natural” dietary supplements with undisclosed synthetic compounds, particularly in categories like weight loss, sexual enhancement, and pain relief [16]. This underscores the need for rigorous evaluation of product content and safety. In the case of berberine hydrochloride, synthetic production (i.e., using synthetic precursors and not plant material) offers several practical advantages, specifically, higher yields [17] and therefore lower production costs, which make it attractive for meeting rising global demand. However, chemical synthesis of berberine raises important safety concerns, specifically the potential for unreacted intermediates.

**Figure 2:**
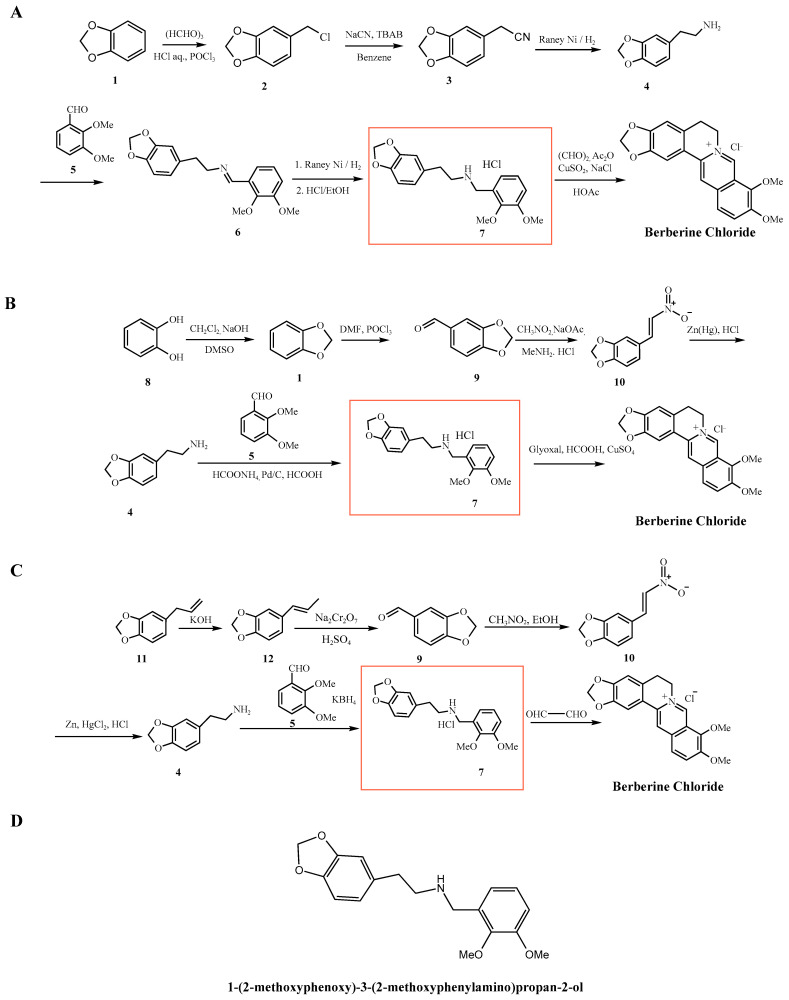
(**A**) Synthetic route described in patent CN113735847A; (**B**) synthetic route described in patent CN106543171A; (**C**) Guangxi Nanning pharmaceutical factory synthetic route; (**D**) chemical structure of 1-(2-methoxyphenoxy)-3-(2-methoxyphenylamino)propan-2-ol.

Among the most serious concerns with synthetic berberine is the potential formation of nitrosamine intermediates. Nitrosamines are genotoxic in several animal models, and some have been classified by the International Agency for Research on Cancer (IARC) as probable or possible human carcinogens [18]. The International Council for Harmonization of Technical Requirements for Pharmaceuticals for Human Use (ICH), Guideline M7 (ICH M7(R2)) guideline (Assessment and Control of DNA Reactive [Mutagenic] Impurities in Pharmaceuticals to Limit Potential Carcinogenic Risk, July 2023) includes nitrosamines in a group known as the “cohort of concern” [19]. The guideline recommends that such impurities be controlled at levels low enough to pose negligible cancer risk in humans, reinforcing the importance of monitoring these compounds during synthesis. Nitrosamines typically form through the reaction of secondary amines with nitrosating agents in acidic environments [20]. Our review found that 10 out of 12 reported synthetic methods involved conditions conducive to nitrosamine formation. For example, synthetic routes described in patents such as CN106543171A (Figure 2A) and CN113735847A (Figure 2B) employ intermediates such as homopiperonylamine (**4**) and aromatic aldehydes (**5**) in the presence of mineral acids (e.g., HCl) to afford the key intermediate 1-(2-methoxyphenoxy)-3-(2-methoxyphenylamino)propan-2-ol (**7**). Similarly, the synthetic route shown in Figure 2C also proceeds via compound **7** as a common intermediate en route to berberine chloride. These reaction conditions, which involve secondary or tertiary amine intermediates under acidic and chlorinating environments, may increase the potential for nitrosamine formation [21,22,23,24].

The broader relevance of this risk is underscored by recent discoveries of nitrosamine contamination in various pharmaceutical products, including Metformin, angiotensin II receptor blockers (ARBs) and proton pump inhibitors (PPIs), which led to recalls and regulatory warnings [25,26,27]. Moreover, nitrosamines have also been detected in dietary supplements including herbal formulations [28,29,30].

In addition to nitrosamines, residual solvents used in synthesis, purification, or crystallization, such as dichloromethane (DCM), benzene, methanol, and acetonitrile pose significant toxicological risks if not adequately removed. Benzene is classified as a Class 1 solvent (to be avoided), while methanol and DCM fall under Class 2 according to the International Council for Harmonisation of Technical Requirements for Pharmaceuticals for Human Use (ICH) guideline Q3C (R8) on Impurities: Residual Solvents (ICH Q3C (R8)), each with strict permitted daily exposure (PDE) limits [31,32]. Additional risks stem from the chemical reagents and catalysts used in berberine synthesis. For instance, some documented processes employ precursors such as catechol and methylene chloride, both of which have recognized carcinogenic potential [33,34,35,36]. These not only pose consumer safety risks but also raise occupational health concerns for manufacturing personnel.

Synthetic ingredients in nutraceuticals, such as berberine, may possibly contain residual impurities like nitrosamines. Increasing awareness of this possibility can help both consumers and businesses make more informed choices. In contrast, berberine obtained through aqueous or organic solvent extraction of plant sources typically avoids the use of nitrosating agents and amine-based intermediates, offering a comparatively safer profile. Natural extracts also often retain a rich phytochemical profile mimicking more closely to that of the natural substance, which may serve as markers of botanical authenticity [37]. Such markers when combined with radiocarbon (Carbon-14) analysis can help solidify natural origin claims and prevent synthetic products from being misrepresented as plant-derived [37].

The goal of this review article is to inform key stakeholders including manufacturers, regulators, and consumers of the risks posed by synthetic routes, and to advocate for a safety-first approach in the production and labeling of berberine-containing supplements.

## 2. Molecular Basis of Nitrosamine Formation

**Figure 3:**
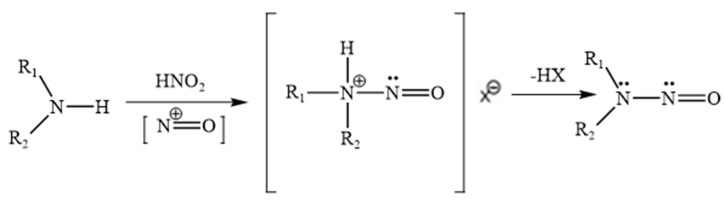
Representative reaction to form nitrosamines.

Nitrosamines are a class of chemical compounds characterized by the presence of a nitroso group (–N=O) bonded to a nitrogen atom, typically of a secondary or tertiary amine (general structure: R_1_N(–R_2_)–N=O). Their formation is primarily driven by nitrosating reactions involving amines and nitrosating agents such as nitrous acid (HNO_2_), nitrosyl chloride (NOCl), dinitrogen trioxide (N_2_O_3_), and other NO^+^-releasing species, as shown in Figure 3. Under acidic conditions, nitrite salts (e.g., NaNO_2_) readily convert to nitrous acid, making them frequent contributors to nitrosamine formation [20].

Secondary amines are the most common precursors for nitrosamine formation due to their ability to form stable *N*-nitroso compounds. In contrast, primary amines typically generate unstable diazonium salts, which rapidly decompose, and tertiary amines may only form nitrosamines through slower dealkylation processes [19,20].

The risk of nitrosamine impurities is a key concern in pharmaceutical and nutraceutical manufacturing, where conditions such as low pH (<5), high temperatures, and the presence of amines or trace nitrites can promote their formation [19,20]. The FDA’s 2024 guidance, control of nitrosamine Impurities in Human Drugs, highlights processes involving secondary or tertiary amines, acid catalysts, or nitrite-containing reagents as particularly high-risk for nitrosamine generation.

Many industrial-scale synthetic routes for berberine hydrochloride reviewed in this work exhibit such features: amino intermediates, use of mineral acids (e.g., HCl) and heating steps, all of which align with FDA-flagged risk factors. If not properly mitigated, these pathways may lead to the formation of nitrosamine impurities in the final product, posing a genotoxic and potentially carcinogenic risk to consumers.

Understanding the chemical mechanisms and identifying at-risk steps in synthetic routes is therefore critical. This includes proactive selection of low-nitrosamine-risk starting materials, controlling pH and temperature during processing, avoiding nitrite contamination, and implementing validated analytical methods for impurity detection.

**Figure 4:**
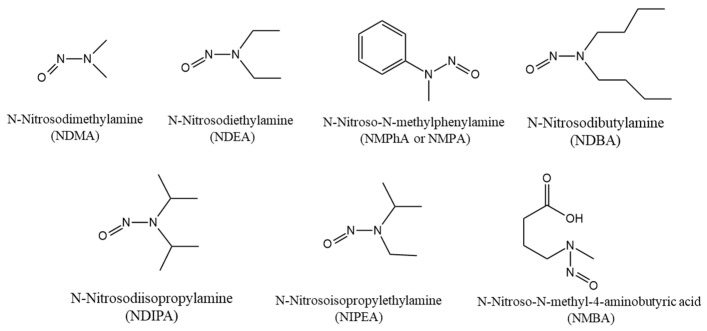
Chemical structures of potential small-molecule nitrosamine impurities.

**Figure 5:**
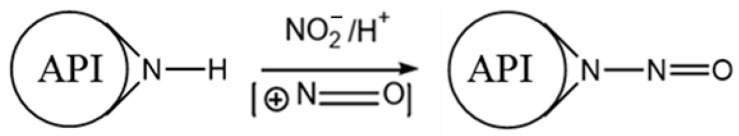
Representative reaction of NDSRI formation.

The FDA classifies nitrosamine impurities into two principal categories: small-molecule nitrosamines (as listed in Figure 4) and nitrosamine drug substance-related impurities (NDSRIs) [20]. Small-molecule nitrosamines, such as NDMA and NDEA, typically form when secondary amines react with nitrosating agents during chemical synthesis or manufacturing processes. In contrast, NDSRIs (illustrated in Figure 5) are structurally linked to a specific active pharmaceutical ingredient (API), often incorporating part of the API within their molecular structure. These impurities arise when the drug substance itself, or a related intermediate, undergoes nitrosation making them generally unique to each API.

While the quaternary isoquinolinium structure of berberine hydrochloride itself is resistant to direct nitrosation, several synthetic intermediates contain secondary amines species. For example, the 1-(2-methoxyphenoxy)-3-(2-methoxyphenylamino)propan-2-ol intermediate (**7**) shown in Figure 2A–C is widely used across multiple industrial routes. Under acidic conditions with trace nitrites, this species could undergo nitrosation to generate an *N*-nitrosamine structurally related to berberine, thus meeting the definition of an NDSRI under FDA and ICH frameworks. The likelihood of such impurities depends on the efficiency of downstream transformations and purification, but their presence underscores the importance of risk-based assessment of both small-molecule nitrosamines and NDSRIs in synthetic berberine production.

## 3. Nitrosamine Risk in Synthetic and Natural Berberine Hydrochloride: Routes, Inte Mediates, and Regulatory Implications

**Figure 6:**
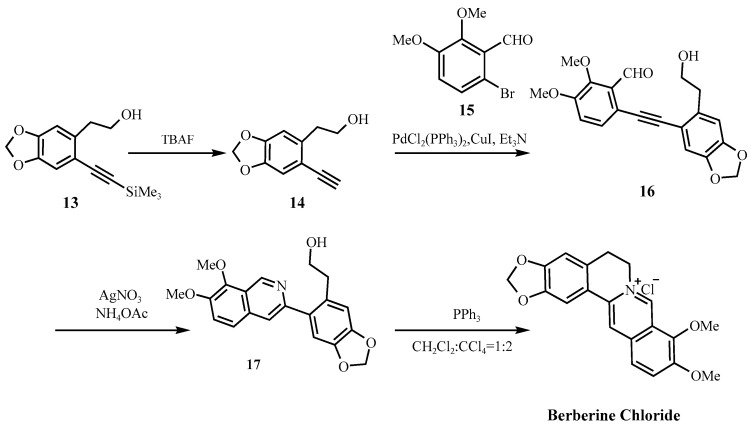
Anand’s synthetic route [40].

**Figure 7:**
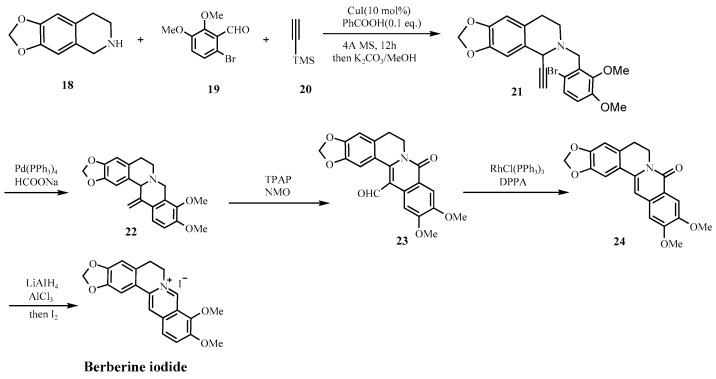
Tong’s synthetic route [41].

Over the past century, synthetic strategies for berberine hydrochloride have evolved significantly, from the foundational condensation reactions reported by Pictet and Gams in 1911 [38], to Kametani’s landmark total synthesis in 1969 [39], and more recently to palladium-catalyzed and convergent approaches (Figure 6 and Figure 7) [40,41]. While these advances have improved yield and efficiency many industrially favored routes continue to utilize reagents and conditions that especially raise safety concerns under current impurity control frameworks, such as ICH M7 (R2) and the FDA’s 2024 guidance on nitrosamine impurities [18,25]. For example, Figure 6 shows a synthesis scheme that employs DCM in the final step, a solvent associated with both environmental and human toxicity concerns, while Figure 7 shows a scheme that involves secondary amine intermediate (**18**), a moiety that can potentially facilitate nitrosamine formation under acidic conditions or in the presence of nitrites.

A cautionary precedent can be drawn from the cases of metformin and ranitidine, both recalled due to contamination with *N*-nitrosodimethylamine (NDMA), a probable human carcinogen [25,26,42]. Investigations revealed that nitrosamines can form when amine-containing intermediates are exposed to trace nitrites under acidic conditions, which are commonly present during synthesis or storage. These nitrites may arise from contaminated solvents, acids, or excipients, illustrating that even tightly controlled processes are vulnerable to inadvertent nitrosation [26].

**Figure 8:**
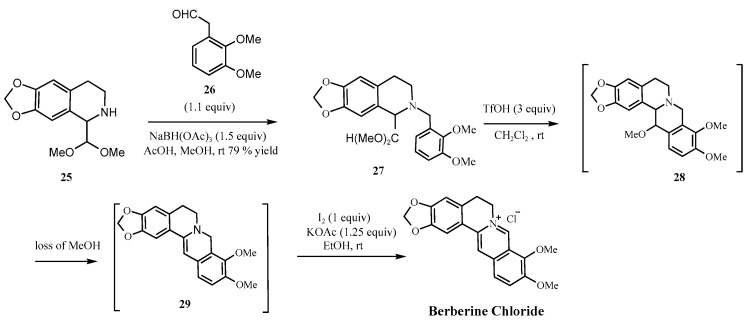
Clift’s synthetic route [43].

Another representative Clift’s synthesis is shown in Figure 8 [43], where berberine hydrochloride is synthesized via a four-step process beginning with the reductive amination of a secondary amine (**25**) and aldehyde (**26**). Although this route avoids classical nitro- sating agents and offers a moderate yield (54%), the presence of the secondary amino group in (**25**) under acidic conditions remains a well-established risk factor.

**Figure 9:**
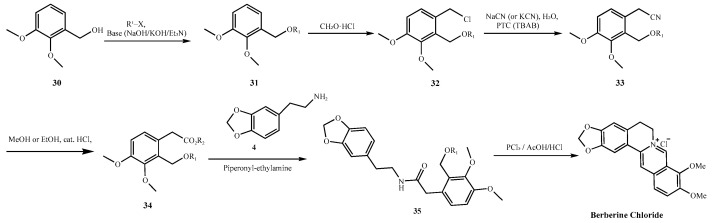
Synthetic route described in patent CN101245064A [44].

**Figure 10:**
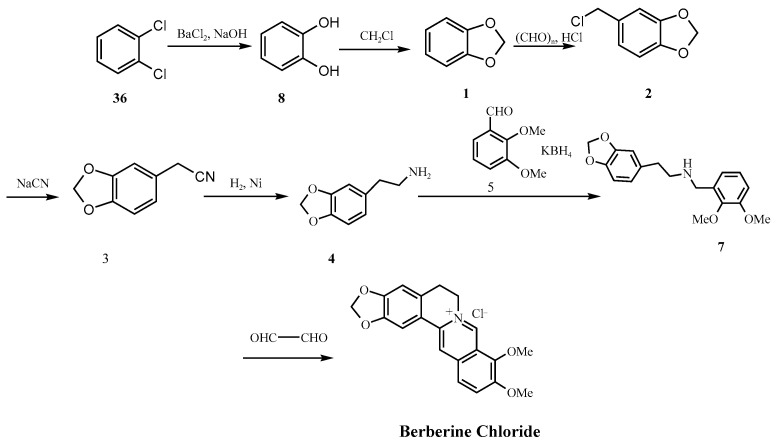
Hangzhou No. 1 pharmaceutical factory synthetic route [45].

Historical and contemporary synthetic routes alike exemplify this risk. For instance, the Guangxi Nanning synthesis (1973) employs zinc amalgam reduction in acidic media (Figure 2C), conditions known to promote nitrosation in the presence of nitrite contaminants. Likewise, the formation of the late-stage intermediate (**35**, Figure 9), as described in patent CN101245064A, proceeds via secondary or tertiary amine intermediates under acidic and chlorinating conditions (e.g., PCl_3_/AcOH·HCl), further increasing the likelihood of nitrosamine formation [44]. Similarly, the first pharmaceutical factory route developed by Hangzhou (Figure 10) involves the formation of 3,4-methylenedioxyphenethylamine (**4**) via sodium cyanide (NaCN) substitution and subsequent hydrogenation. This compound undergoes condensation with aromatic aldehydes (**5**) in the presence of potassium borohydride (KBH_4_) to yield the *β*-amino alcohol intermediate 1-(2-methoxyphenoxy)-3-(2-methoxyphenylamino)propan-2-ol (secondary amine 7), a recurring intermediate common to numerous synthetic schemes [45]. Incomplete reaction or insufficient purification of this intermediate may pose a potential toxicity risk.

Intermediates such as compound **7**, also reported in patents like CN106543171A, are structurally consistent with secondary amines and appear across multiple industrial routes. When exposed to acidic conditions particularly during HCl-mediated condensations or Clemensen-type reductions, such intermediates are vulnerable to nitrosation in the presence of trace nitrites.

According to FDA’s 2024 guidance, three factors facilitate nitrosamine formation: (a) a secondary or tertiary amine, (b) a nitrosating agent (e.g., trace nitrites), and (c) favorable conditions such as low pH and elevated temperature. Many berberine synthesis pathways satisfy at least two of these, even without intentionally added nitrosating agents [20].

Given the synthesis workflow of any nutraceutical that is susceptible to nitrosamines, it becomes crucial to adopt risk-based assessments. High-sensitivity methods such as LC-MS/MS should be employed to ensure residual secondary amines and potential nitrosamines remain below regulatory thresholds (e.g., <96 ng/day for NDMA and <26.5 ng/day for NDEA, per ICH M7) [18].

An analysis of 12 representative synthetic routes for berberine reveals that 10 involve secondary amines or reactive amine intermediates, posing an elevated risk of nitrosamine impurity formation under conditions such as low pH (<5), elevated temperatures, and the presence of trace nitrites, as outlined in the FDA’s 2024 guidance, control of nitrosamine Impurities in Human Drugs [25].

Additionally, several routes involve hazardous reagents or challenging reaction conditions such as use of sodium cyanide, mercury amalgams, or pressurized hydrogenation, all of which introduce additional barriers related to consumer safety and environmental toxicity.

**Figure 11:**
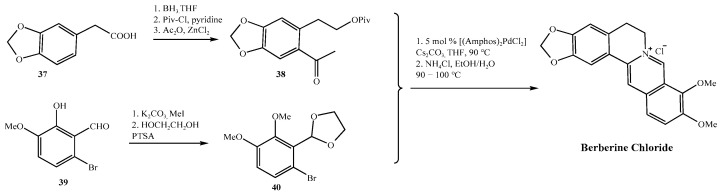
Gatland’s synthetic route [46].

By contrast, newer synthetic strategies reported by Gatland 2014 [46] utilize quaternary ammonium intermediates that are inherently resistant to nitrosation, thereby reducing risk by design (Figure 11). However, these methods depend on expensive palladium catalysts or non-commercial starting materials, which can limit their feasibility for large-scale manufacturing.

These observations underscore the need for route optimization, safer reagent selection, and the adoption of green chemistry principles. Table 1 provides a comparative overview of key reagents, solvents, nitrosamine risk potential, and regulatory considerations across twelve commonly reported synthetic pathways for berberine.

Naturally extracted berberine from plant sources, such as *Berberis aristata* or *Coptis chinensis*, poses minimal risk of nitrosamine contamination because it does not involve synthetic chemical processes where nitrosating agents or amines are typically introduced [47,48,49]. Unlike chemically synthesized pharmaceuticals, the extraction of berberine relies on aqueous or alcohol-based methods that are not conducive to nitrosamine formation. However, such extraction methods give a far less yield compared to synthetic methods. A consolidated overview of synthetic routes, reagents, solvents, and potential nitrosamine risks is provided in Table 1.

**Table 1:** Comparative overview of synthetic routes for berberine hydrochloride: key reagents, toxic hazards, solvents, and nitrosamine risk.

Route/Author	Key Reagents/Intermediates	Toxic or GHS-Classified Reagents	Solvents (ICH Class)	Nitrosamine Risk *
Kametani [39]	Phosphorus oxychloride, diazomethane, formaldehyde	Diazomethane (Acute Tox. 1), POCl_3_ (Skin Corr. 1B)	DCM (Class 2)	Moderate–secondary amine under acid
Guangxi Nanning [23]	Safrole, sodium dichromate, glyoxal	Na_2_Cr_2_O_7_ (Carc. 1B, Muta. 1B), HCl	Aqueous/DCM (Class 2)	High–secondary amine + acid + nitrites
Hangzhou [45]	Catechol, paraformaldehyde, sodium cyanide → β-amino alcohol	NaCN (Acute Tox. 2), Paraformaldehyde, DCM (Carc. 2)	DCM (Class 2)	High–persistent secondary amine + acid
Northeast Pharma [25]	Phenol, paraformaldehyde, methyl chloroacetate	Paraformaldehyde (Acute Tox. 4)	DCM (Class 2)	Moderate–amine intermediates
Gatland [46]	Iodomethane, Pd catalyst, glycol acetal	Iodomethane (Acute Tox. 3), Pd salts	MeOH (Class 2)	Low–late-stage quaternization
Anand [40]	TMS-arylalkyne, silver nitrite, TBAF	AgNO_2_ (Env. Tox. 1), TBAF (Corr.), Pd catalyst	THF (Class 2)	Moderate–nitrite present, no persistent amine
Tong [41]	Copper iodide, TPAP, DPPA	CuI (Aquatic Acute 1), DPPA (Expl. 1.1, Tox. 2)	Acetonitrile (Class 2)	Moderate–amine intermediates
Chen 1 [50]	Catechol, diethyl malonate	–	DCM (Class 2)	Moderate–amine intermediates
Chen 2 [51]	1,2-Methylenedioxybenzene, CuBr·DMS, DMS	CuBr–DMS complex (Irritant), DMS (flammable)	DMS (Class 3)	Moderate–amine intermediates
Clift [43]	Intermediate 14, triflic acid, methanol	TfOH (Corr.), MeOH (Class 2)	Methanol (Class 2)	Low–quaternary ammonium end product
Konno [52]	Boron tribromide, aryl aldehyde	BBr_3_ (Tox. 3, water reactive)	MeOH (Class 2)	Moderate–demethylation + amine
Dong Z. Li [53]	TFAA, benzyl chloride	TFAA (Tox. 3), Benzyl chloride (Carc. 2)	DCM (Class 2)	Moderate–amidation route

Abbreviations: GHS: Globally Harmonized System; Corr.: Corrosive; Carc.: Carcinogenic; Muta.: Mutagenic; Tox.: Toxic. * Nitrosamine Risk categories: Low = No secondary/tertiary amines or nitrosating agents; quaternary salts resistant to nitrosation. Moderate = Secondary/tertiary amines present, but no confirmed nitrosating conditions. High = Secondary/tertiary amines and nitrosating conditions (acidic pH < 5, nitrites, heat). (Aligned with FDA 2024 Guidance and ICH M7 (R2)).

## 4. Residual Solvent Risks in Synthetic Berberine Hydrochloride

Since the synthetic routes, solvents, and hazardous reagents are consolidated into a Table 1, this section provides additional discussion of solvent classification and toxicological considerations not captured fully in tabular form. Organic solvents play a central role in the chemical synthesis and downstream processing of berberine hydrochloride, particularly in steps such as condensation, crystallization, reduction, and purification. However, many of these solvents carry well-characterized toxicological profiles, and their presence in the final product, even in trace amounts, poses potential risks to consumers. According to ICH Q3C(R8) guidelines, solvents are categorized by toxicity and permissible daily exposure limits [31]. As shown in Table 1, several commonly used solvents in berberine synthesis, including dichloromethane, methanol, tetrahydrofuran, and acetonitrile, fall into Class 2, requiring strict control. Others, such as dimethyl sulfide, are Class 3, associated with lower toxicological concern. A systematic evaluation of solvent choice and effective removal strategies is therefore essential to ensure both regulatory compliance and patient safety.

## 5. Natural vs. Synthetic Berberine

Berberine is commonly obtained from *Berberis aristata*, *Berberis vulgaris*, and related species using decoction, acidified water, lime milk treatment, or ethanol extraction. These traditional methods are inexpensive and straightforward, though generally time-consuming and low-yielding [47,48]. Because they rely on physical separation rather than chemical synthesis, they do not generate reactive intermediates such as secondary amines and thus pose minimal risk of nitrosamine formation (Table 2).

Pressurized hot water extraction (PHWE) illustrates this safety profile, employing only hot water at moderate pressure (≈140 °C, 50 bar) for a short time without introducing amine-based reagents [49]. Other solvent extractions (e.g., methanol) may carry minor risks from residual solvents if purification is inadequate, but these risks are still lower than those associated with synthetic routes.

To enhance efficiency, modern green extraction technologies such as microwave-assisted, ultrasonic-assisted, high-pressure, supercritical fluid, pressurized liquid, enzymatic, and aqueous two-phase extraction have been developed [47]. These approaches improve yield, reduce extraction time and solvent use, and help preserve biological activity, although they require specialized equipment and higher costs.

In industry, ethanol and water extractions remain the most widely used due to their simplicity, safety, and food-grade nature, while advanced techniques are increasingly explored for sustainable and reproducible production.

## 6. Discussion

The findings of this review highlight that, although multiple synthetic reaction sequences for berberine hydrochloride have been developed with significant variations in yield, scalability, toxicological burden, and nitrosamine risk, beyond comparing chemistry, these differences have important implications for regulatory compliance, economic feasibility, and consumer confidence. In particular, the persistence of secondary amine intermediates in older routes elevates the risk of nitrosamine drug substance–related impurities (NDSRIs), whereas more modern convergent approaches reduce this concern but still rely on solvents classified by ICH Q3C as Class 2, requiring strict control. Regulatory agencies such as the FDA and EMA increasingly demand robust impurity risk assessments and safer manufacturing practices; meeting these expectations directly impacts the cost of production and the ability to access international markets. At the same time, minimizing nitrosamine risk and adopting greener solvents are not only regulatory obligations, but are also critical to rebuilding consumer trust in light of high-profile recalls of nitrosamine-contaminated medicines.

Furthermore, there are no clinical trials, to the best of our knowledge, that have tested the efficacy and safety of synthetic berberine in humans. On the other hand, there are countless clinical trials that have verified the efficacy and safety profile of naturally sourced berberine based formulation. In contrast, natural berberine, typically extracted from roots of Berberine-containing plant species using solvents like water or ethanol, avoids nitrosamine-prone intermediates and tends to have none or much lower residual toxicity burdens. Natural berberine, a quaternary isoquinolinium alkaloid, exists predominantly as a permanently charged cation (quaternary ammonium structure) [1]. Unlike secondary or tertiary amines, which can undergo nitrosation under acidic or nitrosating conditions to form nitrosamines, berberine lacks a free amine functionality. Its quaternary structure is chemically stable and resistant to nitrosation, even in the presence of nitrite or acidic media. Previous studies indicate that fully substituted quaternary ammonium salts typically have a much lower propensity for nitrosamine formation compared to secondary or tertiary amines, because the nitrogen atom lacks a reactive lone electron pair. However, under certain conditions (e.g., chloramination or radical-mediated degradation), quaternary ammoniums have been shown to act as nitrosamine precursors, though generally at significantly lower yields [54]. Therefore, natural berberine itself considerably decreases a nitrosamine risk; any potential concern arises primarily from synthetic intermediates or precursors used in specific chemical synthesis routes rather than from the naturally occurring alkaloid. Furthermore, natural extracts preserve the broader phytochemical profile of the plant, which may contribute synergistically to bioactivity. Despite challenges such as lower yields, seasonal variability in biomass, and higher processing costs, natural extraction aligns more closely with consumer expectations for transparency, safety, and efficacy. Additionally, natural extracts can preserve the broader phytochemical profile of the plant. Despite challenges such as lower yields, seasonal variability in plant biomass, and higher processing costs, natural extraction aligns more closely with consumer expectations for transparency, safety, and efficacy. To date, there are no reported studies investigating nitrosamines in natural berberine preparations, possibly because the quaternary isoquinolinium structure of berberine hydrochloride confers inherent stability and resistance to nitrosation [1].

The debate between natural and synthetic sourcing in nutraceuticals is not unique to berberine. The curcumin industry has faced similar challenges, where synthetic curcumin, often derived from petrochemicals, has been fraudulently marketed as plant-derived [48]. This has prompted the development of advanced authenticity tools, including carbon-14 radiocarbon dating, isotope ratio analysis, and metabolomic fingerprinting, to verify the botanical origin of curcumin. These tools not only protect consumers from misleading claims but can also help ensure that natural products retain the full spectrum of bioactive compounds. In the case of berberine, the same concerns apply; synthetic versions lack the complex chemical fingerprint of the plant matrix and may carry undetected impurities like nitrosamines or residual solvents. To address these issues, some natural berberine formulations, such as HIMABERB^®^, have been evaluated using ^14^C isotope labeling study to verify their botanical origin, thereby providing an additional means of authenticity assessment [55].

As the industry moves forward, several key recommendations can help close these safety gaps. Manufacturers synthesizing nutraceuticals using chemical pathways that could be susceptible to nitrosamine formation should conduct formal nitrosamine risk assessments as per ICH M7, and regularly audit synthetic pathways to eliminate high-risk reagents or steps. Regulatory authorities should mandate clear labeling of berberine origin, synthetic or natural, to enhance consumer trust and enforce transparency.

In summary, while synthetic berberine is a cheaper alternative, it must meet rigorous impurity control standards to be considered safe for long-term human use. Natural berberine, though more costly, presents fewer safety concerns. By learning from cases like curcumin and adopting science-driven authentication and quality control, the nutraceutical sector can ensure that berberine products, regardless of source are safe, effective, and transparent to the end consumer.

## 7. Conclusions

The increasing use of bioactive products by the industrial syntheses in the nutraceutical sector, exemplified by the case of berberine highlights a critical gap in transparency and safety. While synthetic berberine hydrochloride offers advantages in scalability and cost, it presents significant concerns related to nitrosamine formation and residual solvent contamination.

In contrast, naturally extracted berberine from botanical sources aligns more closely with clean-label expectations and demonstrates a lower toxicological burden. Although its production is costlier, natural berberine provides a safer, more holistic alternative, especially important in a health-conscious consumer market increasingly demanding authenticity transparency and sustainability.

To ensure the long-term safety and credibility of berberine-containing products, the nutraceutical industry must adopt a more rigorous and science-driven approach. To support long term safety, synthetic manufacturing of nutraceuticals should proactively address the potential for toxic impurities, particularly nitrosamine contamination, by optimizing synthesis workflows and minimizing the use of high-risk reagents. Additionally, to support natural origin claims, manufacturers should implement advanced source verification methods such as radiocarbon dating. Lessons from the curcumin industry should serve as a cautionary framework: when synthetic analogs are introduced without adequate safeguards, consumer trust is compromised, and long-term risks may be eliminated.

## Figures and Tables

**Table 2:** Comparative overview of natural vs. synthetic berberine.

Parameter	Natural Berberine (Plant-Derived)	Synthetic Berberine (Chemically Synthesized)
Source	Extracted from medicinal plants (*Berberis*, *Coptis*) with centuries of traditional use	Produced via multistep chemical synthesis using petrochemical-derived precursors
Apparatus	Stainless-steel extraction vessels, percolators, or Soxhlet apparatus; concentration typically by rotary evaporator or spray dryer	Glass-lined or stainless-steel reactors with reflux condensers; purification through crystallization or chromatography
Yield	Low, derived from renewable, biogenic sources	High, dependent on synthetic efficiency and raw material availability
Purity	Requires tedious purification, but typically free from synthetic byproducts	High assay purity, but may harbor trace-level synthetic impurities such as nitrosamines
Toxic Impurity Risk	Minimal; aqueous or alcoholic extraction avoids nitrosamine formation	Elevated; potential for nitrosamines, residual solvents, or unreacted intermediates
Nitrosamine Risk (FDA 2024 Guidance)	Negligible under aqueous, alcoholic and acid-base extraction conditions	Notable concern: many synthetic routes can lead to nitrosamine formation unless mitigated
Process Solvents	Water, Ethanol, methanol, environmentally benign (Except methanol) and food-grade	Involves organic solvents (e.g., dichloromethane, toluene), which may require stringent residue control
Consumer Perception	Viewed as holistic, natural, and safer for long-term use	Increasing concern over “lab-made” compounds and hidden risks among informed consumers
Environmental Impact	Lower carbon and chemical footprint if sustainably harvested	Higher impact unless green chemistry principles and solvent recovery are employed
Cost Efficiency	Higher per-gram cost, but offset by holistic composition and perceived safety	Lower cost per gram, but potential trade-offs in safety and public trust

## Data Availability

No new data was created or analyzed in this study. Data sharing is not applicable to this article.
